# *Echinops spinosissimus* Turra Root Methanolic Extract: Characterization of the Bioactive Components and Relative Wound Healing, Antimicrobial and Antioxidant Properties

**DOI:** 10.3390/plants11243440

**Published:** 2022-12-09

**Authors:** Saida Hanane Zitouni-Nourine, Nabila Belyagoubi-Benhammou, Fatima El-Houaria Zitouni-Haouar, Omar Douahi, Faouzia Chenafi, Habiba Fetati, Siham Chabane Sari, Assia Benmahieddine, Chahinez Zaoui, Fatima Zohra Nadjet Mekaouche, Fawzia Atik Bekkara, Nadia Kambouche, Angelo Gismondi, Houari Toumi

**Affiliations:** 1 Pharmaceutical Development Research Laboratory, Department of Pharmacy, Faculty of Medicine, Oran 1 Ahmed Ben Bella University, Oran 31000, Algeria; 2 Natural Products Laboratory, Department of Biology, Faculty of Natural and Life Sciences, Earth and Universe, University Abou-Bekr Belkaïd, Tlemcen 13000, Algeria; 3 Laboratory of Biology of Microorganisms and Biotechnology, Department of Biotechnology, Faculty of Natural and Life Sciences, Oran 1 Ahmed Ben Bella University, Oran 31000, Algeria; 4 Microbiology Laboratory, Department of Pharmacy, Faculty of Medicine, University Abou-Bekr Belkaïd, Tlemcen 13000, Algeria; 5 Department of Sociology, Faculty of Social Sciences, University Oran 2, Oran 31000, Algeria; 6 Anatompathological Department, Faculty of Medicine, Oran 1 Ahmed Ben Bella University, Oran 31000, Algeria; 7 Developmental and Differentiation Biology Laboratory, Faculty of Natural and Life Sciences, Oran 1 Ahmed Ben Bella University, Oran 31000, Algeria; 8 Organic Synthesis Laboratory, Department of Chemistry, Faculty of Exact and Applied Sciences, Oran 1 Ahmed Ben Bella University, Oran 31000, Algeria; 9 Laboratory of General Botany, Department of Biology, University of Rome Tor Vergata, 00133 Rome, Italy

**Keywords:** thorny globe thistle, phyto complex, liquid chromatography, antiradical power, antibiotic effect, wound-healing activity

## Abstract

*Echinops spinosissimus* Turra subsp. *bovei* (Asteraceae) is a medicinal plant in western Algeria. Traditionally, roots and inflorescences are employed as hypertensive agents and in the treatment of hemorrhoids. The current study evaluates the chemical composition, antioxidant, antimicrobial, and wound-healing properties of the root methanolic extract from *E. spinosissimus* subsp. *bovei*. The content of total phenolics, flavonoids, and tannins was determined. In addition, the phenolic profile was typified. The studied plant extract resulted in being primarily composed of Apigenin, Kaempferol, and their derivatives. The total phenolic content was equal to 95.31 ± 2.90 mg GAE/g DW, while the number of flavonoids and condensed tannins was 16.01 ± 0.16 mg CE/g DW and 8.30 ± 0.65 mg CE/g DW, respectively. The methanolic extract was found to exhibit antioxidant activity towards the DPPH radical, with an IC_50_ of 7.99 ± 0.28 mg/mL and a TAC of 30.30 ± 0.54 mg AAE/g DW, as well as an antibacterial effect, especially against *P. aeruginosa*. No significant wound-healing property was observed, even though the histopathological observations showed enhanced wound-healing quality. According to our evidence, *E. spinosissimus* could represent a source of phytochemicals with potential beneficial effects for human health in terms of antioxidant and antibiotic properties, although further investigations on this species are needed.

## 1. Introduction

*Echinops* L. genus belongs to Asteraceae, a plant family that is dispersed all over the world and includes more than 120 species [1]. In Algeria, this genus is commonly represented by *E. spinosus* L. [2,3], usually known as the synonym of *E. spinosissimus* Turra (thorny globe thistle). According to Quezel and Santa [4], *E. spinosus* presents two subspecies: *E. spinosus* ssp. *spinosus* Maire (var. Chaetocephalus Pomel) and *E. spinosus* ssp. *bovei* (Boiss.) Maire (var. Pallens Maire), also known as *E. bovei* Boiss. 

Literature on the phytochemistry of this species is limited; chemical investigations on *E. spinosissimus* have revealed a Phyto complex that includes metabolites relative to different molecular classes. Fokialakis et al. [5] reported in this species the presence of two thiophenes compounds, the α-Terthiophene and the Acetylene 2,2-dimethyl-4-[5’-(prop-1-ynyl)-2,2’-bithiophen-5-yl]-1,3-dioxalane, while another group [6,7] isolated three novel sesquiterpenoids (Echinopine A, Echinopine B, 11-Hydroxyisocom-2-en-5-one) from the methanol extract of *E. spinosissimus* root grown in Morocco. An Egyptian study documented the existence of flavonoids (e.g., Apigenin; Apigenin-7-*O*-β-glucopyranoside or cosmosiin; Apigenin-7-β-D-*O*-(6″-*O*-p-coumaroyl)-glucopyranoside) in the aerial parts of *E. spinosissimus*, although even two quinoline alkaloids, the Echinopsine and the Echinorine, have been identified in the inflorescences [8,9]. Two further research studies on the same plant detected the presence of 22 other flavonoids, such as Hesperetin (39.233 mg/100 g) and Hesperidin (34.589 mg/100 g), which were the major phenolic components of the aerial districts [10], and 13 sterols, such as β-Sitosterol (44.97%) and Stigmasterol (34.95%) [11]. Apigenin-7-*O*-β-D-glucoside-(4″-*O*-*trans*-*p*-coumaroyl) and a C30-Pentacyclic triterpadiene A-neooleana-3(5),12-diene have been also identified in the same plant [12]. Finally, in *E. spinosissimus* aerial parts, nine triterpenoids, specifically one oleanane-type, four taraxastane-types, three lupane-types, and one phytosterol, have been also isolated [13]. 

Based on this premise, it is clear that even if *E. spinosissimus* is a very interesting species, other studies are needed to characterize its Phyto complex and relative biological properties. Hence, the present work was conducted to investigate the phenolic content and profile of *E. spinosissimus* root methanolic extract and assess its bioactive potential, in terms of antioxidant, antimicrobial, and wound-healing effects. In detail, according to our knowledge, this is the first report showing the wound-healing capacity and an HPLC-PDA-ESI-MS analysis of the methanolic extract from E. spinosissimus root.

## 2. Results and Discussion

### 2.1. Phenolic Profile

**Figure 1:**
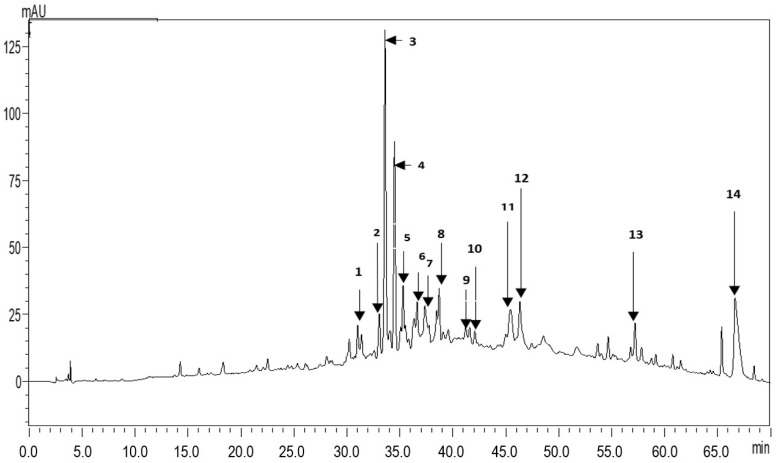
UV chromatogram of *E. spinosissimus* root methanol extract at 280 nm.

HPLC UV-chromatograms of the identified compounds and their retention times, maximum absorption wavelengths, and MS spectral data are illustrated in Figure 1 and Table 1. Fourteen compounds were recorded. Apigenin and its derivatives (peaks no. 3, 4, 5, 6, 12) were the most abundant compounds. We also observed the presence of Kaempferol and its derivatives (peaks no. 7, 11, 14), Eriodictyol-4’-O-neohesperidoside-7-O-glucoside (peak no. 8), Isorhamnetin-3-O-rutinoside (peak no. 13), three phenolic acids, that is Chlorogenic, Cinnamic, and Caffeic acids (peaks no. 1, 2, 9), Quercetin-3-galactoside.

It should be noted that the flavonoids isolated from the whole plant were mainly flavones. Apigenin is the most common flavonoid aglycone and the literature has documented its presence in different species of the *Echinops* genus (*E. niveus* Wall. ex Wall, *Echinops echinatus* Roxb, *Echinops integrifolius* Kar. & Kir *and Echinops albicaulis* Kar. & Kir) supporting our evidence [14,15,16,17]. 

### 2.2. Total Phenolic, Flavonoid and Condensed Tannin Content

The results of total phenolic, flavonoid, and condensed tannin contents are reported in Table 2. The amount of total phenolics was 95.31 ± 2.90 (mg GAE/g DW), while the levels of flavonoids and condensed tannins were 16.01 ± 0.16 mg CE/g DW and 8.30 ± 0.65 mg CE/g DW, respectively. Our results were in accordance with those reported by Kheder et al. [18] about the polyphenolic contents in *E. spinosus* L root from Tunisia. In that work, the results showed that polyphenolics could vary considerably according to solvent polarity. Another work focused on the aerial part of *E. spinosissimus* Turra from the northwest of Algeria mentioned that the total phenolics were significantly higher than those from roots. Indeed, their content was equal to 36.1 ± 0.0005 mg GAE/100 g DM and 16.1 ± 0.001 mg GAE/100 g DM, respectively, in the two districts. In parallel, the flavonoid content in the aerial part was estimated at 13.37 ± 0.001 mg CE/100 g DM, while in roots, their level was 3-fold lower (4.78 ± 0.0005 mg EC/100 g of DM) [19]. The quantitation of total phenolics, tannins, and total flavonoids in the air-dried thorny globe thistle by Al-Harbi et al. [20] indicated concentrations of 66.54 mg GAE/100 g DM, 20.28 mg CE/100 g DM, and 44.6 CE/100 g DM, respectively. Samir et al. [21] investigated the composition of the aerial parts of *E. spinosus* L from Egypt, registering an amount of total phenolics equal to 0.23 ± 2.43 % (*w*/*w*), flavonoids at 0.004 ± 0.04%, and tannins at 2.88 ± 0.07%. 

The phytochemical screening of *E. spinosus* L inflorescence extracts from Tunisia reported that methanol was the best solvent for isolating phenols (895.14 mg GAE/g), while the ethyl acetate for flavonoids (215.36 mg QE/g) [22].

### 2.3. Antioxidant Activity

The results of the antioxidant activity assessed in the methanolic root extract by two tests were reported in Table 2. DPPH IC_50_ value of the *E. spinosissimus* extract was 7.99 ± 0.28 mg/mL, while TAC was estimated at 30.30 ± 0.54 mg AAE/g DW. Our quantifications were low compared to those documented in Khedher et al. [18], who showed that thorny globe thistle root ethanolic extract had a strong capacity to reduce DPPH radicals (IC_50_ value of 147 μg/mL). In the same way, Gheffour et al. [19] verified that the tannin extract of *E. spinosus* aerial parts had a higher capacity to scavenge DPPH radicals (8.25 μg/mL) than the root extract (23 μg/mL). Comparatively, the methanolic extract of *E. giganteus* root showed an in vitro free radical scavenging effect of 12.54 mg equivalent weight of Trolox per 100 g [23], while the methanolic extract from *E. orientalis* seeds and leaves revealed DPPH antiradical activity of 60% at 40 µg/mL, although its isolated constituents (i.e., β-Sitosterol,1-methyl quinoline-4(1H)-one; Apigenin-7-*O*-β-D-glucoside; Aapigenin-7-*O*-(6″-transp-coumaroyl-β-D-glucopyranoside)) did not exhibit an important antioxidant potential [24]. 

### 2.4. Antimicrobial Activity

As reported in Table 3, *S. aureus* appeared very resistant to the different tested concentrations (i.e., 100, 200, and 400 mg/mL) of *E. spinosissimus* root crude extract. On the other hand, we observed moderate antimicrobial activity towards the other two bacterial strains, especially against *P. aeruginosa* at the highest concentrations (i.e., 200 and 400 mg/mL). Thus, the plant extract seemed effective in inhibiting the growth of Gram-bacteria, including *P. aeruginosa,* which is strongly involved in wound infection and plays an important role in the development of wound chronicity. Indeed, wound-colonizing bacteria such as *P. aeruginosa* delay wound healing, being capable of degrading skin proteins and inhibiting fibroblast growth [25].

According to the literature, the extracts of the aerial parts of *E. spinosissimus* from the Egyptian Mediterranean coast have recorded moderate antibacterial activity at 6 mg/mL against *E. coli, Bacillus cereus*, and *S. aureus*, with an inhibition diameter of 12 mm. This activity increased significantly at higher concentrations, reaching the maximum activity against *B. cereus* (42 mm) [26]. Preparations obtained using plant material (leaves) from Saudi Arabia have revealed, at the concentration of 100 mg/mL, diameters of inhibition varied between 09.5 and 13.5 mm for all tested strains [27]. The same methanolic extracts have shown relatively low antifungal effects against *Botrytis cinerea* and *Fusarium solani,* moderate activity against *Alternaria alternata*, but high inhibition potential against *Stemphylium botryosum* [28]. Surprisingly, *E. spinosissimus* air-dried extracts have revealed low antimicrobial power against *S. aureus* (13 mm) and no activity against *E. coli* and *C. albicans* [29]. Table 3 presents the MIC values of the root methanolic extract from *E. spinosissimus* against the tested bacteria. The plant sample had MIC values of 25 mg/mL for both *P. aeruginosa* and *S. aureus*, while for *E. coli*, it showed 50 ± 0 mg/mL. Our results agreed with those reported by Mothana et al. [30]. which confirmed that the methanolic and hot aqueous extracts from *E. spinosissimus* did not show any interesting activity against three bacterial strains, *S. aureus, B. subtilis*, and *M. flavus*, with MIC values varying between 500 and 1000 μg/mL. Similarly, the isolated fractions from the *E. spinosus* hexane extract did not exhibit significant antibacterial activity, presenting MIC values of 125.0 μg/mL against *S. aureus*, *B. cereus*, and *M. luteus* (for the latter MIC > 125.0 μg/mL) and no substantial antifungal activity [11]. Worthy of note is that *E. amplexicaulis* Oliv root methanolic extract has also shown a hopeful effect against the *M. tuberculosis* multidrug-resistant strain with a MIC of 50 µg/mL [31].

### 2.5. Wound-Healing Activity

**Figure 2:**
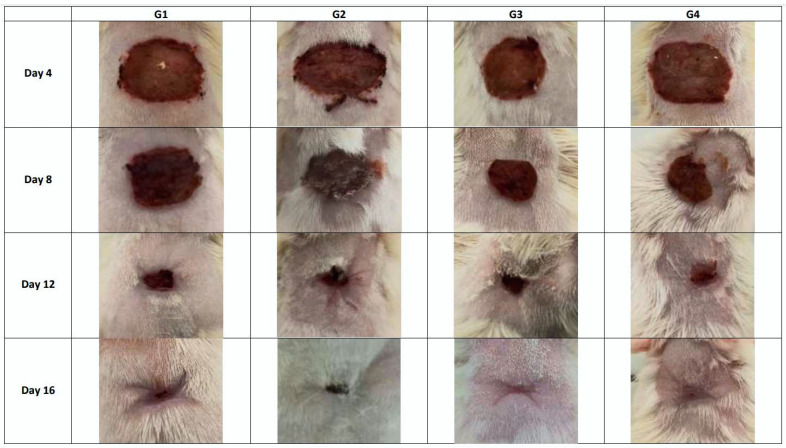
Morphological representation of wound contraction from control and test groups at days 4 and 16 after the excision.

**Figure 3:**
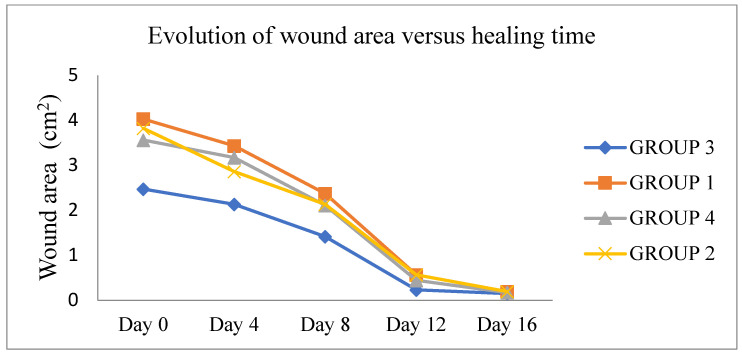
Regression curve of the wounded area on the days of measurement for each treatment. G1—MEBO containing 0.25% of β-sitosterol; G2—negative control; G3—white petrolatum (vehicle); and G4—*E. spinosissimus* methanolic extract ointment (MEO).

**Figure 4:**
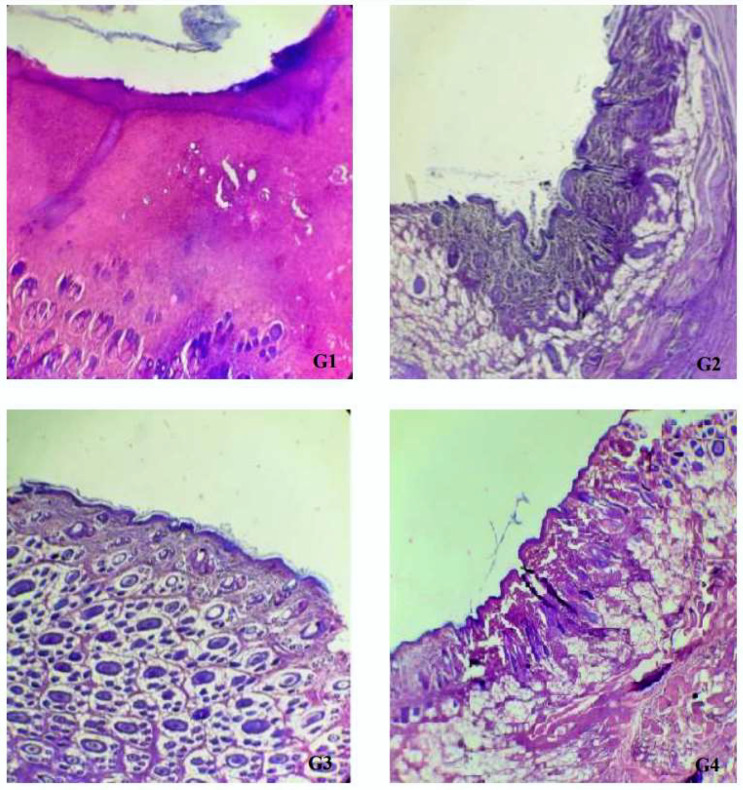
Histological sections observed by photonic microscope (100×) of the skin tissues obtained from day 16 after excision wound (100×). (**G1**) (positive control MEBO^®^): Keratinized acanthotic epidermis, neoangiogenesis, presence of some neutrophils testifying moderate inflammation, polymorphic inflammatory infiltrates, collagen in small quantities with moderate fibrosis. (**G2**) (negative control; without any treatment): Superficial epidermis with normal structure, no inflammation, and a more pronounced presence of collagen. (**G3**) (white petrolatum): Epidermis of regular thickness, keratinized, surmounting by a conjunctivovascular dermis, comprising some inflammatory elements, sebaceous glands, and hair follicles; absence of fibrosis and presence of a normal deposit of collagen. (**G4**) (MEO, *E. spinosissimus* extract): Epidermis of regular thickness, keratinized, surmounting a connective and vascular dermis comprising ancillary structures, such as sebaceous glands and hair follicles. Presence of a few lymphocytes and small blood vessels. Collagen of moderate density and regular fibrosis was also noticed.

The results of dimensions and contraction rates of the wound areas, calculated at 4, 8, 12, and 16 days, are reported in Table 4. Macroscopic observations of the excision wounds, as illustrated in Figure 2, indicated that the topical application of MEO did not significantly increase the wound contraction rate in comparison with the controls. All experimental groups showed a progressive decrease in the wound surface area from day 0 until day 16 (Figure 3). In particular, during this period, in the vehicle-treated group, the wound contraction rate ranged from 12.17% to 94.00%, whereas in MEO rats, there was a recovery from 9.83% to 95.00%, with a complete closure by the end of the experiment (15 ± 0.53 days). In the MEBO^®^ (β-sitosterol)-positive control group, the percentage varied from 15.33% to 95.5.%, while the negative control group revealed contraction percentages of 25.33% to 94.67%. It can be concluded that MEO had wound-healing activity closer to MEBO^®^ since there was no significant difference in the contraction percentages on days 12 and 16 for all four groups. (Table 4). Comparing our results to the only study that has investigated the wound-healing properties of the extract obtained from the aerial parts of *E. spinosus* L (Saudi Arabia), we found that our extract exhibited better healing activity with respect to that mentioned above, with a wound area of 0.17 ± 0.06 on day 16 versus 0.21 ± 0.06 on day 20 measured for the Saudi species [20]. To confirm our previous result of wound-healing activity, histological analysis of the skin tissue on day 16 was realized (Figure 4). MEO-treated wound areas were filled with fibrous connective tissue, an abundant amount of mature oriented fibroblasts, and well-organized dense bundles of collagen fibers, composing a mature collagenous matrix. The wound area was completely covered by epidermal cells with a structure very similar to that belonging to normal skin. The presence of cells in full mitosis at the level of the epidermis, which was very well structured, was observed. The presence of keratinocytes was also detected. Ancillary structures of the skin, such as pilosebaceous glands, were strongly represented in the sections. It was noticeable from the anatomopathological analysis of the animal sections that the *E. spinosissimus* methanol extract might exhibit a pro-healing action in the excision wound model by favoring cellular proliferation, collagen deposition, and re-epithelialization.

The correlation among phytoconstituents, biological activities, and wound-healing properties is complex and hard to be defined. Indeed, peculiar dynamic mechanisms involved in the reproduction of damaged tissues and requiring accurate coordination of connective tissue restoration, angiogenesis, and re-epithelialization are triggered by injuries [32]. For instance, epithelialization is a process that involves both the proliferation and migration of epithelial cells across the wounded bed [33]. It has been reported that the wound-healing effect of any tested drugs can be considered proportional to the re-epithelialization period and enhanced wound contraction [34]. The presence of free radicals at or around the wound bed may postpone the wound-healing process through the annihilation of lipids, proteins, collagen, proteoglycans, and hyaluronic acid. Phytoconstituents that manifest significant antioxidant activity may, therefore, preserve tissue viability and ameliorate wound healing [35].

In research aimed at identifying the bacterial pathogens present in infected wounds from 213 patients, the most common strains were *S. aureus* (37%), *P. aeruginosa* (17%), *Proteus mirabilis* (10%), *E. coli* (6%), and *Corynebacterium* spp. (5%) [36]. Wound infection plays an important role in the development of chronicity, delaying wound healing [37]; thus, the application of antibacterial substances would seem essential for an ideal healing process [38]. It is documented that polyphenols may act by changing cell membrane permeability and, consequently, become excellent natural antimicrobial metabolites [39]. Consequently, the *E. spinosissimus* methanolic extract, being rich in these secondary metabolites (as testified in our study) and also in flavonoids (i.e., Apigenin derivatives), might enhance the quality of the wound-healing procedure. In addition, flavonoids and tannins have been considered promoters for wound contraction, due to their astringent, antimicrobial, antioxidant, anti-inflammatory, antiallergic, angiogenic, and fibroblast-related proliferation properties [40]. In particular, Apigenin, Kaempferol, and their glycosides have been reported to have wound-healing properties [41,42]. It has been also stated that Kaempferol-3-*O*-glucoside from *Ipomoea carnea* Jacq. increased collagen deposition, hydroxyproline content in the granulation tissue, and fibroblast proliferation [43]. According to Clericuzio et al. [44], Kaempferol-3-*O*-[(6-caffeoyl)-b-glucopyranosyl-(1-3) a-rhamnopyranoside]-7-*O*-α-rhamnopyranoside might accelerate keratinocyte cell migration. In another study, rats treated with a 1% (*w*/*w*) Kaempferol ointment solution manifested the utmost healing effect in diabetic excisional and non-diabetic incisional wounds [45]. In this context, Apigenin has been proven to act on collagenase activity [46], exert an anti-inflammatory property [47], and attenuate acute lung injury in mice by decreasing the production of pro-inflammatory cytokines (IL-6, IL-1β, and TNF-α) through the inhibition of COX-2 and NF-κB activation pathways [48,49]. In addition, analgesic and anti-inflammatory potential have been linked to Apigenin due to its inhibitory effect on PGE2, TNF-α, and pro-inflammatory cytokines, such as IL-1β and IL-6, in mouse and rat models of inflammatory diseases [50]. Choi et al. [51] have reported both in vitro and clinical efficacy of Apigenin in restoring and protecting the viability of normal human dermal fibroblasts exposed to ultraviolet (UV) radiation, together with the capacity to decrease collagenase and matrix metalloproteinase (MMP)-1 expression. Apigenin-7-*O*-glucoside and Schaftoside have been also documented to induce the expression of collagen type III mRNA, suggesting that they can stimulate collagen production via different mechanisms [52]. According to Tu et al. [53], Apigenin promotes the angiogenic factor by modulating the Caveolin-1 signaling pathway. Finally, Zain et al. [54] have documented that flavonoid C-glycosides (i.e., Orientin, Isoorientin, Vitexin, and Isovitexin) possess useful biological properties, such as antioxidant and wound-healing effects, showing the ability to induce fibroblast proliferation and migration.

## 3. Materials and Methods

### 3.1. Plant Material and Extraction Method

**Figure 5:**
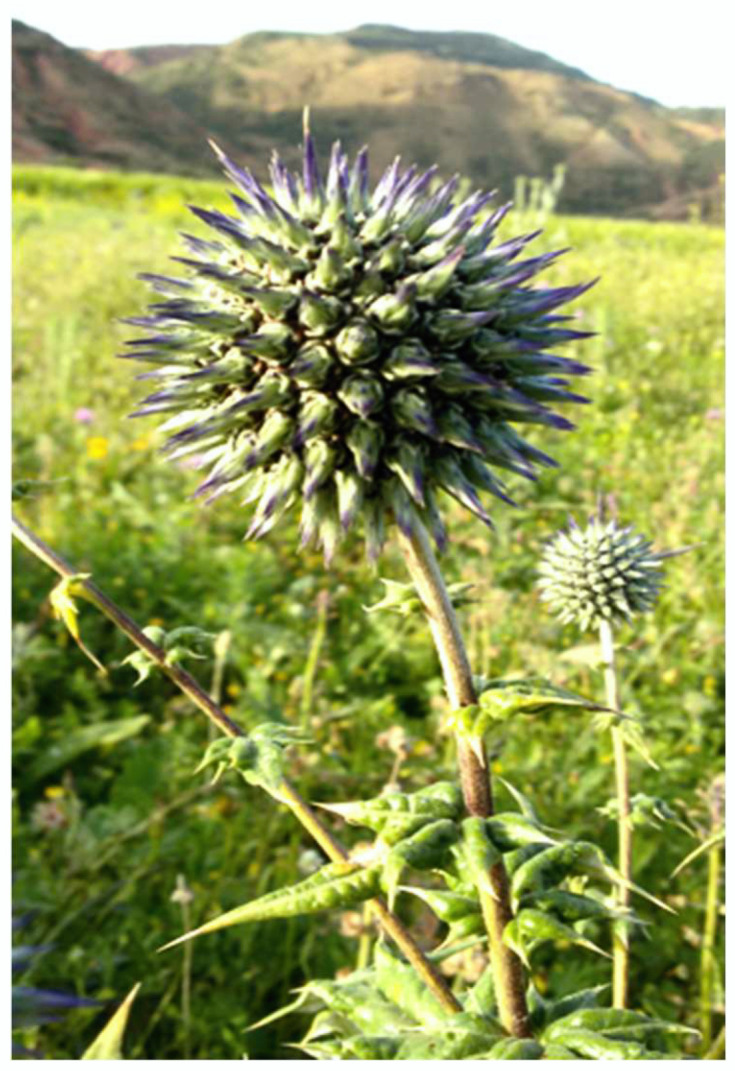
*Echinops spinosissimus* Turra (Sidi Belattar Mostaganem, 2018).

Fresh roots of *E. spinosissimus* (Figure 5) were collected in April 2018 from plants growing naturally in western Algeria (Sidi Belattar Mostaganem, 363 Km west of Algiers). The identification and authentication of the plant species were carried out by Ms. Nador Hayat, a botanist at the Medical Botany Laboratory of Pharmacy Department, University Oran 1 (Algeria), where a voucher specimen (Essb 0026) has been deposited. Roots were air-dried and ground to a fine powder. Sixty grams of this plant material were subjected to successive extraction according to Ni et al. [7] with slight modifications. In detail, the procedure was performed in a continuous extraction apparatus (Soxhlet) with solvents of increasing polarity, for 3 h each, in the following order: Petroleum ether, ethyl acetate, and methanol. The residue left after each extraction was air-dried before extraction with the next solvent. The methanol extract was filtered through Whatman filter paper No. 1.

### 3.2. Phenolic Profiling by HPLC-PDA-ESI-MS

Chromatographic separation was carried out on a Thermo Finnigan Surveyor Plus HPLC apparatus equipped with a quaternary pump, a Surveyor UV-Vis photodiode array (PDA) detector, and an LCQ Advantage max ion trap mass spectrometer (MS; Shimadzu Prominence-i/LC-2030C 3D), coupled via an electrospray ionization (ESI) source. The analysis was carried out on a SUPELCO C18 (25 cm × 4.6 mm, 5 µm). The solvent system was made of acidified water (pH = 3) with acetic acid (solvent A) and acidified methanol (pH = 3) with acetic acid (solvent B). A gradient elution from 10 to 100% B in 50 min was used. The flow rate was 0.8 mL/min [55] and the injection volume was 5 µL. The chromatograms were obtained at 280 nm. HPLC-PDA-ESI-MS analyses of the phenolics were carried out using the ESI interface, in the negative ion mode. ESI conditions were as follows: Temperature: 350 °C; nebulizer pressure: 35 psi; N_2_ drying gas-flow rate: 10 L/min; fragmentor voltage: 135 V; capillary voltage: 4500 V; mass spectral range: 100–1.600 *m*/*z* [56].

### 3.3. Total Phenolic Content

Total phenolics were determined using the Folin–Ciocalteu procedure, as described by Singleton and Ross’s method [57]. A volume of 200 µL of the extract was mixed with 1 mL of the Folin-–Ciocalteu reagent and diluted 10 times with water and 0.8 mL of 7.5% sodium carbonate solution. After stirring, 30 min later, the absorbance was measured at 765 nm. Gallic acid was used as a standard for the calibration curve. The total phenolic content was expressed as milligrams of Gallic acid equivalents per gram of dry matter (mg GAE/g DM). 

### 3.4. Total Flavonoid Contents

The total flavonoid content was determined by a colorimetric assay using the method described by Zhishenet al [58]. Briefly, 500 µL of the Catechin standard solution at different concentrations or plant extract was mixed with 1500 µL of distilled water and 150 µL of 5% (*w*/*v*) NaNO_2_ solution at time zero. After 5 min, 150 µL of 10% AlCl_3_ (*w*/*v*) was added and incubated for 6 min at room temperature. Then, 500 µL of NaOH (1 M) was added. Immediately, the mixture was completely agitated to homogenize the content. The absorbance of the solution was measured at 510 nm against a blank. The total flavonoid content was expressed as mg of Catechin equivalents per gram of dry matter (mg CE/g DM).

### 3.5. Condensed Tannin Content

Proanthocyanidins were measured using the vanillin assay described by Julkunen-Titto [59]. One thousand-five hundred microliters of the vanillin/methanol solution (4%; *w*/*v*) were added to 50 µL of each extract. Then, 750 µL of HCl was added and the sample was incubated at room temperature for 20 min. The absorbance at 550 nm was measured against a blank. The amount of total condensed tannins was expressed as milligrams of Catechin equivalents per gram of dry matter (mg CE/g DM), using a calibration curve. 

### 3.6. Total Antioxidant Capacity

The total antioxidant capacity was determined according to the phosphomolybdenum method of Prieto et al. [60]. An aliquot (0.3 mL) of the sample was mixed with 3 mL of the standard reagent (0.6 M sulfuric acid; 28 mM sodium phosphate; 4 mM ammonium molybdate). Then, the reaction mixture was incubated at 95 °C for 90 min. After the mixture had cooled to room temperature, the absorbance was measured at 695 nm. Total antioxidant capacity was expressed as milligrams of Ascorbic acid equivalents per gram of dry matter (mg AAE/g DM) and as milligrams of Gallic acid equivalents per gram of dry matter (mg GAE/g DM), using adequate calibration curves.

### 3.7. DPPH Assay

The hydrogen atom donation ability of the phenolic compounds was measured as a scavenging effect against the 2,2-diphenyl-1-picrylhydrazil free radical (DPPH) [61]. Fifty microliters of the plant extract were added to 1950 µL of a 0.025 g/L DPPH methanol solution. After 30 min of incubation at room temperature, the absorbance was read against a blank at 515 nm. DPPH free radical scavenging activity was calculated as a percentage (%), using the following formula:

DPPH scavenging activity (%) = (A_blank_ − A_sample_/A_blank_) × 100

where A_blank_ is the absorbance of the control reaction (containing all reagents except the test compound) and A_sample_ is the absorbance of the test compound. 

The concentration of the extract providing 50% inhibition of the DPPH radical (IC_50_) was extrapolated by linear regression from the plotted graph that reports the inhibition percentage against extract concentrations. The ascorbic acid was used as a positive control.

### 3.8. Antimicrobial Activity

The plant extract was tested on three bacterial species, including the Gram-positive *Staphylococcus aureus* (ATCC 25923), the Gram-negative *Escherichia coli* (ATCC 25922), and *Pseudomonas aeruginosa* (ATCC 27853). The stocks were revivified, and turbidity was adjusted to 0.5 McFarland, which corresponds to 1.2 × 10^8^ UFC/mL (O.D. = 0.08 to 0.1/λ = 625 nm) [62].

#### 3.8.1. Agar Disc Diffusion Method

Bacterial inhibition effects were determined according to the disc diffusion method, recommended by the Clinical and Laboratory Standards Institute (CLSI) guidelines [63]. Bacterial suspensions were prepared in Mueller Hinton Broth (MHB) and then incubated at 37 °C for 24 h. The tested microorganism suspension was adjusted to an optical density of 0.5 McFarland (10^8^ CFU/mL). Then, the suspension of each culture was spread on the solid medium plates using a sterile cotton swab. Whatman No.1 sterile filter paper discs (6 mm in diameter) were impregnated with 5, 10, and 20 µL of plant methanolic extract. Standard antibiotic disks (75 µg Ticarcilline and 10 U Penicillin G) were used as positive controls to control the sensitivity of the tested microorganism. The plates were left for 2 h at 4 °C and then kept at 37 °C for 24 h. After the incubation period, the inhibition zone diameters (IZDs) were measured, including the paper disk (in mm).

#### 3.8.2. MIC Determination

The minimum inhibitory concentration (MIC) of *E. spinosissimus* extract was determined using the micro broth dilution method [64] according to the protocols of the Clinical and Laboratory Standards Institute (CLSI). All tests were performed in Mueller Hinton Broth and cultures of each strain were prepared overnight. Microorganism suspensions were adjusted in a spectrophotometer to a final density of 10^6^ CFU/mL. Then, a two-fold dilution was carried out in a 96-well microplate to obtain dilutions of the extracts with concentrations ranging from 0.019 to 5 mg/mL. An equal volume of the microbial inoculum from the tested strains grown in overnight broth culture was added to the final concentration of 5 × 10^5^ CFU/mL per well. After incubation for 18–24 h at 37 °C in a normal atmosphere, the MIC was defined as the lowest concentration of the extract at which the microorganisms did not exhibit visible growth. The growth of the microorganisms was detected by turbidity. MIC values were expressed in µg/mL. 

### 3.9. In Vivo Wound Healing Activity

The experiment was performed according to the International Guiding Principles for Biomedical Research Involving Animals (1990) and to the Algerian 98–11 Law of 22 August 1998. Female Wistar rats (208.71 ± 22.43 g) obtained from the Pasteur Institute of Algiers were used for the study. They were kept in individual cages, respecting the nyctemeral cycle and under standard conditions of temperature and humidity. They were fed with commercial rat feed and water ad-lib. Animals were divided into four groups with six animals in each group (*n* = 6). MEBO^®^ cream, Vehicle (ointment base), and plant treatment (2% methanolic plant extract in ointment base prepared by trituration method using white petrolatum [65]; MEO) were applied to animals’ wounds for 16 days [66]. The animals were anesthetized with 3–5% light ether, before the creation of the wound. An excision wound was inflicted by cutting away the skin measuring 1.5 × 1.5 cm^2^ in diameter at a 2 mm depth at the dorsal cervical region of each animal from a predetermined shaved area [67]. Hemostasis was accomplished by blemishing the wound with a cotton swab drowned in a normal saline solution. The wounded animals were housed separately in different cages. The wounds were left undressed in an open environment and animals were carefully observed during the 16 days of treatment regarding their general appearance, checking if there was the presence or absence of bleeding, exudate, and crust. Animals showing any sign of infection were separated, excluded from the study, and replaced. The wound area was measured instantly by placing a transparent tracing paper over the wound and tracing it out using a permanent marker [34,67]. The tracing paper was then canned to calculate the wound surface areas (WSA) with the software AUTOCAD.

The wound area was estimated on different days (0th, 4th, 8th, 12th, and 16th days) and the percentage of wound contraction was calculated as shown below [68]:

$$\%\;\mathrm{Wound}\;\mathrm{contraction}=\frac{\mathrm{Wound}\;\mathrm{area}\;\mathrm{on}\;\mathrm{day}\;0-\mathrm{Wound}\;\mathrm{area}\;\mathrm{on}\;\mathrm{day}\;n\;*\;100}{\mathrm{Wound}\;\mathrm{area}\;\mathrm{on}\;\mathrm{day}\;0}$$

The period of epithelialization was reckoned as the number of days needed for the dead tissue remnants to fall off without any residual raw wound [69]. The determination of the epithelialization period was carried out for all wounded animals. Group 1 was topically treated with MEBO^®^ containing 0.25% β-Sitosterol (positive control); Group 2 was left untreated (negative control); Group 3 received topical application of white petrolatum (vehicle); Group 4 received topical application of MEO once per day, until the sacrifice. On the 16th post-operative day, the excision skin tissues from all animals were processed for the histopathological examination. In all cases, the dissection was carried out at the end of the healing phase. All samples were fixed in 10% buffered formalin (pH 7.0) for at least 24 h, blocked with paraffin, sectioned into 5 µm thicknesses, and then stained with the hematoxylin-eosin reagent. Histological slides were analyzed under an optical microscope to observe the inflammatory and scarring process. To check the quality of wound healing, the following histological parameters were considered: The number of infiltrated inflammatory cells (i.e., polymorphonuclear cells, mononuclear cells), vascular proliferation, fibroblastic proliferation, reepithelialization, and collagen deposition [70].

### 3.10. Statistical Analysis

All analyses were carried out in triplicate (independent biological replicates). Data were presented as the mean ± standard deviation. MicrocalOrigin 6 and Microsoft Excel 2003 were used for statistical and graphical evaluations. A one-way analysis of variance (ANOVA) test was performed by SPSS 25 to evaluate the statistical significance of the data between the 4 animal groups; *p* < 0.05 was considered statistically significant. 

## 4. Conclusions

In summary, the methanolic extract of *E. spinosissimus* root from western Algeria was found to exhibit antioxidant activity and antibacterial effects against Gram-negative bacteria, especially *P. aeruginosa*. Based on its polyphenolic content, characterized here by spectrophotometric and HPLC-MS investigations, and on its biological properties, thorny globe thistle root extract could be used as an ingredient for pharmaceutical products or a source of phytochemicals showing beneficial potential for human health, including infection prevention and wound-healing power.

## Figures and Tables

**Table 1:** Phenolic components detected in the *E. spinosissimus* root methanolic extract by HPLC-PDA-ESI-MS.

Peak Number	Rt (min)	UV (λ max)	Molecular Ion [M-H]–(*m*/*z*)	Identified Compound
1	31.020	201–327	353	Chlorogenic acid
2	33.064	273	149	Cinnamic acid
3	33.607	271–335	593–271	Apigenin-6,8-glucoside
4	34.498	271–333	431–271	Apigenin 7-*O*-glucoside
5	35.310	272–334	431	Apigenin-8-C-glucoside
6	36.627	271–332	431–283	Apigenin-6-C-glucoside
7	37.369	268–295–315	739–285	Kaempferol *p*-coumaroyl-diglycosided
8	38.709	273–322	757	Eriodictyol-4’-*O*-neohesperidoside-7-*O*-glucoside
9	41.595	269–299–327	295	Caffeic acid derivative
10	42.082	269–296–349	463	Quercetin-3-galactoside
11	45.436	269–327	785–285	Kaempferol 3-*O*-acyldiglycoside
12	46.336	268–332	269	Apigenin
13	57.204	295–344	623	Isorhamnetin-3-*O*-rutinoside
14	66.645	197–275–315	901–285	Kaempferol-7-*O*-rhamnosyl-glucoside

Several authors have investigated the Phyto complex of the aerial parts from *E. spinosissimus* Turra; a chemical analysis has shown the presence of 22 flavonoids, including Hespirtin (39.233 mg/100 g), Hesperidin (34.589 mg/100 g), Luteolin-6- arabinose-8-glucose (25.344 mg/100 g), Apigenin-6-arabinose-8-galactose (23.049 mg/100 g) and Apigenin-6-glucose-8-rhamnose (20.083 mg/100 g), which were the main metabolites [9], while another work also identified Apigenin, Apigenin-7-*O*-β-glucopyranoside (cosmosiin), and apigenin-7-β-D-O-(6″-*O*-E-p-coumaroyl)-glucopyranoside [10]. In addition, a new apigenin derivative, named Apigenin-7-*O*-β-D-glucoside-(4″-*O*-*trans*-*p*-coumaroyl), was reported [12].

**Table 2:** Quantitative estimation of the polyphenolic contents, total antioxidant capacity, and IC_50_ concentration in the DPPH test.

	Total Phenolics (mg GAE/g DW)	Total Flavonoids (mg CE/g DW)	Condensed Tannins (mg CE/g DW)	TAC (mg AAE/g DW)	IC_50_ (DPPH) (mg/mL)
Root methanolic extract	95.31 ± 2.90	16.01 ± 0.16	8.30 ± 0.65	30.30 ± 0.54	7.99 ± 0.28
Ascorbic Acid	/	/	/	/	0.090 ± 0.002

**Table 3:** Antibacterial activity of the methanolic extract from *E. spinosissimus* root.

Concentration (mg/mL)	Inhibition Diameters (mm)		
	*E. coli*	*P. aeruginosa*	*S. aureus*
100	7	0 ± 0	0 ± 0
200	7	10.66 ± 0.58	0 ± 0
400	8	12.33 ± 0.58	0 ± 0
Positive control	27 ± 0.82 (Ticarciline)	27.33 ± 0.47 (Ticarciline)	34 ± 0.82 (Penicillin G)
MIC (mg/mL)	50 ± 0	25 ± 0	25 ± 0

**Table 4:** Effect of topical treatment by MEO and standards on wound contraction after 16-day excision wound in rats.

Experimental Group	Wound Area (cm^2^) and Percentage of Wound Contraction					Epithelization Period (Days)
	Day 0	Day 4	Day 8	Day 12	Day 16	
Positive control (MEBO^®^) (G1)	4.03 ± 0.70 *^G3^	3.43 ± 0.76 *^G3^ (15.33%)	2.37 ± 0.40 *^G3^ (41.00%)	0.56 ± 0.31 (86.50%)	0.19 ± 0.12 (95.5%)	11.50 ± 1.80
Negative control (untreated group) (G2)	3.82 ± 0.33	2.86 ± 0.48 (25.33%)	2.13 ± 0.48 *^G3^ (44.17%)	0.52 ± 0.53 (85.33%)	0.19 ± 0.18 (94.67%)	12.33 ± 1.88
Ointment base control (G3)	2.47 ± 0.36 *^G1,G4,G2^	2.13 ± 0.51 *^G1,G4^ (12.17%)	1.41 ± 0.12 *^G1,G2^ (41.83%)	0.23 ± 0.05 (90.17%)	0.15 ± 0.09 (94.00%)	11 ± 1
MEO 2% (G4)	3.56 ± 0.77 *^G3^	3.17 ± 0.42 *^G3^ (9.83%)	2.11 ± 0.5 (40.83%)	0.44 ± 0.12 (87.50%)	0.17 ± 0.06 (95.00%)	11.83 ± 1.07

Statistical significance was determined by a one-way ANOVA test followed by Bonferroni’s post-test. The percentage of wound contraction in the tested group was calculated and compared to the standard groups. Values are expressed as means ± S.E. *(n* = 6 in each group), * significantly different from the mentioned group at *p* < 0.05.

## Abbreviations

DPPH	2,2-Diphényl-1- Picrylhydrazyl
HPLC-PDA-ESI-MS	High-Performance Liquid Chromatography Photo Diode Array Mass Spectrometry.
IC50	Inhibition Concentration 50
MIC	Minimum Inhibitory Concentration
MEO	Methanolic Extract Ointment.
TAC	Total Antioxidant Capacity
